# First Evidence for Internal Ribosomal Entry Sites in Diverse Fungal Virus Genomes

**DOI:** 10.1128/mBio.02350-17

**Published:** 2018-03-20

**Authors:** Sotaro Chiba, Atif Jamal, Nobuhiro Suzuki

**Affiliations:** a Institute of Plant Science and Resources, Okayama University, Kurashiki, Okayama, Japan; b Asian Satellite Campuses Institute, Nagoya University, Nagoya, Aichi, Japan; c Graduate School of Bioagricultural Sciences, Nagoya University, Nagoya, Aichi, Japan; d National Agricultural Research Centre, Islamabad, Pakistan; National Institutes of Health

**Keywords:** dsRNA virus, hypovirus, internal ribosome entry site, mycovirus, noncanonical translation

## Abstract

In contrast to well-established internal ribosomal entry site (IRES)-mediated translational initiation in animals and plants, no IRESs were established in fungal viral or cellular RNAs. To identify IRES elements in mycoviruses, we developed a luciferase-based dual-reporter detection system in *Cryphonectria parasitica*, a model filamentous fungus for virus-host interactions. A bicistronic construct entails a codon-optimized *Renilla* and firefly luciferase (*ORluc* and *OFluc*, respectively) gene, between which potential IRES sequences can be inserted. In this system, ORluc serves as an internal control, while OFluc represents IRES activity. Virus sequences in the 5′ untranslated regions (UTRs) of the genomes of diverse positive-sense single-stranded RNA and double-stranded RNA (dsRNA) viruses were analyzed. The results show relatively high IRES activities for Cryphonectria hypovirus 1 (CHV1) and CHV2 and faint but measurable activity for CHV3. The weak IRES signal of CHV3 may be explained by its monocistronic nature, differing from the bicistronic nature of CHV1 and CHV2. This would allow these three hypoviruses to have similar rates of translation of replication-associated protein per viral mRNA molecule. The importance of 24 5′-proximal codons of CHV1 as well as the 5′ UTR for IRES function was confirmed. Furthermore, victoriviruses and chrysoviruses tested IRES positive, whereas mycoreoviruses, partitiviruses, and quadriviruses showed similar Fluc activities as the negative controls. Overall, this study represents the first development of an IRES identification system in filamentous fungi based on the codon-optimized dual-luciferase assay and provides evidence for IRESs in filamentous fungi.

## IMPORTANCE

Cap-independent, internal ribosomal entry site (IRES)-mediated translational initiation is often used by virus mRNAs and infrequently by cellular mRNAs in animals and plants. However, no IRESs have been established in fungal virus RNAs or cellular RNAs in filamentous fungi. Here, we report the development of a dual-luciferase assay system and measurement of the IRES activities of fungal RNA viruses in a model filamentous fungal host, *Cryphonectria parasitica*. Viruses identified as IRES positive include hypoviruses (positive-sense RNA viruses, members of the expanded *Picornavirus* supergroup), totiviruses (nonsegmented dsRNA viruses), and chrysoviruses (tetrasegmented dsRNA viruses). No IRES activities were observed in the 5′ untranslated regions of mycoreoviruses (11-segmented dsRNA viruses), quadriviruses (tetrasegmented dsRNA viruses), or partitiviruses (bisegmented dsRNA viruses). This study provides the first evidence for IRES activities in diverse RNA viruses in filamentous fungi and is a first step toward identifying *trans*-acting host factors and *cis*-regulatory viral RNA elements.

## INTRODUCTION

Eukaryotic translation involves the initiation, elongation, termination, and ribosome recycling steps (1, 2). Each step, particularly the initiation step, has been extensively studied and is known to be regulated by many factors working in a well-coordinated manner. There are two modes for the initiation step: the cap-dependent and -independent mechanisms. The first comprises the recognition of the cap structure (m^7^GpppX) of mRNA by eukaryotic initiation factor 4F (eIF4F), loading of the 43S preinitiation complex, and scanning toward the initiation codon (1). The second one entails the entry of ribosomes into internal mRNA sites with the aid of *cis* elements and *trans*-acting factors. Most eukaryotic mRNAs employ the canonical cap-dependent scanning mechanism for protein synthesis. Many RNA viruses, largely positive-sense RNA viruses, along with a minor portion of cellular mRNAs, utilize cap-independent internal ribosomal entry site (IRES)-mediated initiation.

An IRES was first identified in the 5′ untranslated regions (UTRs) of picornaviruses and later in other viruses, mostly positive-sense single-stranded RNA (ssRNA) viruses, as exemplified by the initially identified poliovirus (PV) and encephalomyocarditis virus (EMCV) (3, 4). A recent systematic high-throughput analysis estimated that ~10% of human cellular mRNAs employ IRESs (5). However, strong evidence is generally unavailable for many IRESs of cellular mRNAs (6). Depending on sizes, required host factors, and structural features, viral IRESs are generally classified into three to five groups (1, 7–9). For example, the poliovirus IRES of ~450 nucleotides (nt), a member of class I, requires the most initiation factors and likely has a highly structured conformation, while the intergenic IRES of dicistroviruses with a size of ~200 nt, a member of class IV, requires no initiation factors. In addition to initiation factors, specific IRES *trans*-acting factors (ITAF) are required. Furthermore, there seem to be another class of IRESs containing unstructured sequences with shorter motifs that may be able to bind ITAF or 18S rRNA (5). However, no universal sequence motif unique to IRESs has been identified (10), making it difficult to predict IRESs based on sequence information. Cell-type- and host-specific differences in ITAF expression levels and the affinity of orthologous initiation factors for IRESs may determine levels of IRES-mediated translation, tissue tropism, and host range (11).

An increasing number of viruses have been discovered in major groups of fungi, including double-stranded RNA (dsRNA) viruses, positive-strand (+) and negative-strand (−) ssRNA viruses, and single-stranded DNA (ssDNA) viruses (12–16). Predominantly, these viruses have an RNA genome, largely dsRNA genomes. Studies of these viruses have enhanced our knowledge about virus diversity in terms of genome structures, virion morphology, and host-virus interactions. Fungal viruses are now classified into at least 16 virus families, although many remain unassigned. However, little is known about their expression strategies, except for a few viruses. Mycoreovirus mRNAs have cap structures at their 5′ termini, as in the case of other reoviruses (17, 18), and appear to follow canonical translation. Members of the genus *Totivirus* infecting *Saccharomyces cerevisiae* steal cap structures from cellular mRNAs via the cap-snatching activity of their capsid protein (CP) (19, 20). This activity has not yet been confirmed in members of other genera within the family *Totiviridae*. Rather, IRESs have been identified in the 5′ portion of mRNAs of a giardiavirus and leishmaniavirus, protozoan-infecting members of the family *Totiviridae* (21, 22). However, many fungal RNA viruses are hypothesized to utilize unusual initiation strategies regardless of their genome type. This hypothesis is supported by the fact that multiple minicistrons are found in the relatively long 5′ UTRs of viral transcripts. For example, the prototype hypovirus Cryphonectria hypovirus 1 (CHV1), which is one of the best-studied viruses of filamentous fungi, has an approximately 500-nt-long 5′ UTR with 7 mini-open reading frames (mini-ORFs). The well-studied victoriviruses Helminthosporium victoriae virus 190S (HvV190S) and Rosellinia necatrix victorivirus 1 (RnVV1) have 289-nt and 372-nt 5′ UTRs with 2 and 1 mini-ORF, respectively (23–25); these features are usually observed in IRESs. Although there are a few online tools for IRES prediction, accurate identification requires experimental substantiation.

*Cryphonectria parasitica*, a phytopathogenic ascomycete, has been established as a model filamentous fungus for exploring virus-virus and virus-host interactions (26). This fungus can support the replication of many homologous and heterologous viruses. Multiple transformation can be readily achieved, and viral RNA and virion introduction can be performed. A single-luciferase assay system, though not highly sensitive, was developed earlier to identify stop/restart translational regulation in CHV1 (27). In this study, we developed a dual-reporter system using codon-optimized firefly and *Renilla* luciferase genes. By applying this technology, we identified IRESs in many fungal RNA viruses in *C. parasitica*.

## RESULTS

### Codon-optimized luciferase genes provide higher levels of chemiluminescence.

**FIG 1:**
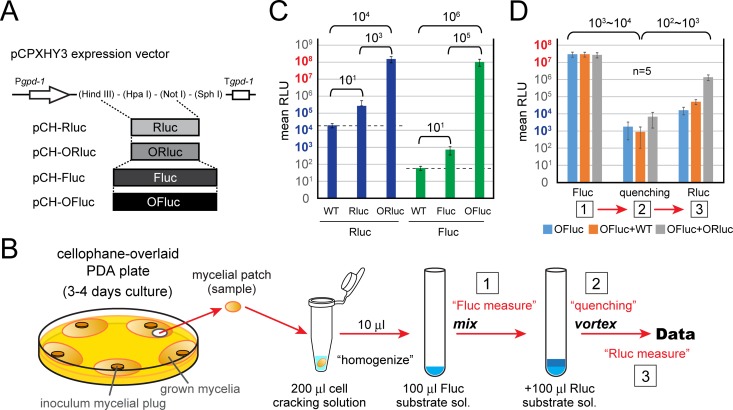
Development of the dual-luciferase (DL) reporter system in *C. parasitica*. (A) Construction of transformation vectors expressing luciferase genes. Original or codon-optimized *Renilla* and firefly luciferase genes (*Rluc* and *Fluc* or *ORluc* and *OFluc*, respectively) were cloned into the pCPXHY3 vector, and pCH-Rluc, pCH-Fluc, pCH-ORluc, and pCH-OFluc were obtained. The wild-type (WT) *C. parasitica* EP155 standard strain was transformed with these constructs and served as a negative control (dotted lines in C). (B) Schematic representation of the dual-luciferase reporter assay performed in this study. (C) Effectiveness of codon-optimized luciferase genes in *C. parasitica*. Luciferase activities were measured for transformants and a nontransformed WT strain as a reference. The raw relative luminescence units (RLU) as integrated values in 12 s are presented. (D) Confirmation of the dual-luciferase system in *C. parasitica*. Mycelia of WT or ORluc- or OFluc-expressing lines were homogenized. The OFluc-expressing line (OFluc, half diluted with PBS), a mixture of WT and OFluc-expressing lines (OFluc+WT), or a mixture of OFluc- and ORluc-expressing lines (OFluc+ORluc) was subjected to analysis. RLU were measured at three time points: 1, firefly luciferase measurement; 2, quenching step by addition of *Renilla* firefly buffer; and 3, *Renilla* luciferase measurement by addition of the substrate for *Renilla* luciferase, as shown in panel B.

The original firefly and *Renilla* luciferase genes (*Fluc* and *Rluc*, respectively) and codon-optimized firefly and *Renilla* luciferase genes (*OFluc*, a modified *Oluc* gene, and *ORluc*, respectively) (see Fig. S1 in the supplemental material) were compared regarding the chemiluminescence level in *C. parasitica*. These genes were transgenically expressed from a fungal expression vector with a hygromycin resistance gene as a selectable marker, pCPXHY3 (pCH-Rluc, -ORluc, -Fluc, and -OFluc) (Fig. 1A). Ten individual transformants were grown on cellophane-overlaid potato dextrose agar (PDA) plates, and mycelia were subjected to a reporter assay (Fig. 1B). The nontransformed (wild-type [WT]) samples showed a low level of background for firefly luciferase (~10^2^ relative luminescence units [RLU]), whereas a relatively high level of background was observed for *Renilla* luciferase (~10^4^ RLU) (Fig. 1C, dashed line). A significant increase of firefly luciferase activity by codon optimization was observed (~10^5^-fold, Fluc versus OFluc). Similarly, ORluc showed greater chemiluminescence than did the original Rluc (~10^3^-fold), indicating that these codon optimizations were highly effective in *C. parasitica* (Fig. 1C). Importantly, the abovementioned background of *Renilla* luciferase was negligible because of the high activity of ORluc (Fig. 1C, WT versus ORluc, ~10^4^-fold difference). Moreover, a preliminary experiment for the development of the dual-luciferase (DL) assay was carried out using mixtures of homogenates derived from independent OFluc and ORluc transformants and the WT strain (Fig. 1D). Chemiluminescence by OFluc was quenched well after mixing with *Renilla* luciferase buffer, in the absence of its substrate (Fig. 1D, steps 1 and 2). When the substrate was added to this suspension, chemiluminescence by ORluc was specifically obtained at a high level; the mixture of OFluc and ORluc showed at least a 100-fold-higher level than the negative control, OFluc plus WT (Fig. 1D, step 3). Hence, these results provide a foundation for the dual-luciferase assay in filamentous fungi with OFluc and ORluc.

**FIG 2:**
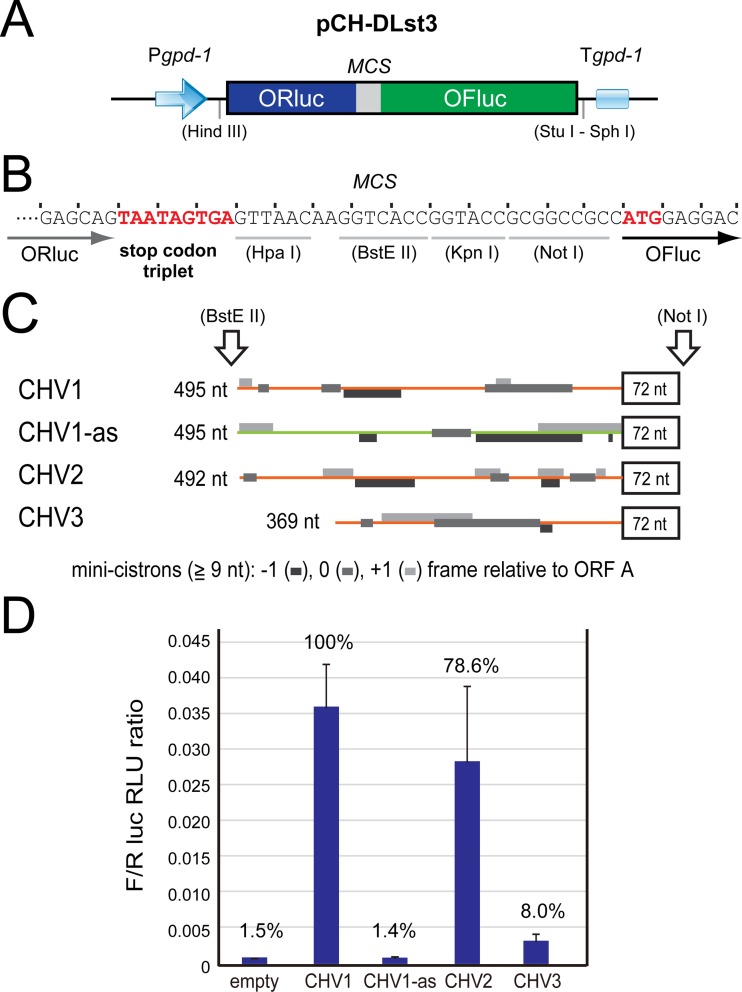
Detection of hypovirus IRES elements. (A and B) Schematic representation of the IRES identification foundation construct, pCH-DLst3. *ORluc* and *OFluc* genes are tandemly inserted in the pCPXHY3 expression vector, under the control of the *gpd-1* promoter and terminator (A). The *ORluc* gene is terminated with a stop codon triplet. A multiple cloning site (MCS) is created between the luciferase genes (B). (C and D) IRES detection by dual-luciferase (DL) reporter assays. *C. parasitica* hypovirus-originating sequences were analyzed. The 5′ untranslated region (UTR) and the adjacent 72 nt from the 5′-proximal ORF region of CHV1, CHV2, and CHV3 were cloned in pCH-DLst3 (C). The 72-nt sequences are in frame to the downstream *OFluc* gene. The antisense sequence of the CHV1 5′ UTR was used as a negative control (CHV1-as). The DL assay with 1 s of measurement was conducted, and the ratio of OFluc RLU to ORluc RLU (OFluc RLU standardized by ORluc RLU [F/R ratio]) was calculated and graphically shown (D). Open boxes, coding regions; black lines, plasmid sequences; orange lines, viral UTRs; light green line, antisense of CHV1 UTR; gray boxes, small cistrons (consisting of over 9 nt) on 5′ UTRs in three different frames.

10.1128/mBio.02350-17.1 FIG S1  Sequences of codon-optimized luciferase genes. (A) Sequence of the *OFluc* gene. The sequence was modified from the original *Oluc* gene (63) such that the BstEII recognition site was abolished by nucleotide substitutions indicated by red letters. (B) Sequence of the *ORluc* gene. The triple stop codons inserted into the bicistronic dual-luciferase foundation construct, pCH-DLst3 (Fig. 2B), are underlined. Download FIG S1, PDF file, 0.1 MB. Copyright © 2018 Chiba et al. 2018 Chiba et al. This content is distributed under the terms of the Creative Commons Attribution 4.0 International license.

### Development of a dual-luciferase assay system for the identification of IRESs in *C. parasitica*.

Bicistronic, dual-luciferase assay systems are necessary for the identification of IRESs. A dual-luciferase (DL) gene cassette was inserted between the HindIII and SphI recognition sites of pCPXHY3 to prepare a foundation construct, pCH-DLst3 (Fig. 2A). The plasmid has a multicloning site between *ORluc* and *OFluc* for the insertion of IRES candidates and a stop codon triplet for the translation termination of *ORluc* (Fig. 2B). It should be noted that nontransformants (WT) gave 10^2^ and 10^4^ RLU as backgrounds (Fig. 1C). Consequently, OFluc activities in transformants with this construct were estimated to be 10^4^ RLU, which was higher by approximately 10^2^-fold than the WT nontransformants. The unexpected OFluc activities shown in the transformants by pCH-DLst3 may be attributable to possible degradation of transgene transcripts, a cryptic promoter in the *ORluc* sequence, or stop/restart translation. The same level of OFluc activity was also observed for transformants with viral sequences that are believed to lack IRESs (see below). Importantly, the difference in OFluc activities between pCH-DLst3 and pCH-DL, a variant without the stop codon triplet for *ORluc* producing the OFluc-ORluc fusion protein, was great enough to test IRES candidate viral sequences and identify IRESs (Fig. S2).

10.1128/mBio.02350-17.2 FIG S2  Toward the development of a dual-luciferase-based IRES detection system in *C. parasitica*. (A) Construction of pCPXHY3-based foundation vectors. The plasmid pCH-DL has no stop codon for *ORluc*, while pCH-DLst3 has triple stop codons (asterisks) for *ORluc*. The downstream MCS and OFluc regions are identical in these clones, and the ORluc and OFluc coding regions are in frame. (B) Comparison of mean chemiluminescence values for pCH-DL variants. *C. parasitica* strains transformed with two pCH-DL variants were subjected to the DL assay. The mean RLU (2 s of integrated values) are presented. Although a detectable background of the OFluc RLU was recognized in pCH-DLst3, this was 10^3^ lower than that of pCH-DL and small enough to test potential IRES sequences and measure IRES activities (OFluc RLU). Luciferase activities were measured for a nontransformed WT strain as a reference. Download FIG S2, TIF file, 0.7 MB. Copyright © 2018 Chiba et al. 2018 Chiba et al. This content is distributed under the terms of the Creative Commons Attribution 4.0 International license.

### IRES activities in hypoviral sequences.

In total, 12 viral sequences were tested in this study and are summarized in Table 1. The list includes dsRNA viruses (five families) to (+) ssRNA viruses (one family). We first tested the untranslated regions of three representative hypoviruses (CHV1 to CHV3) with (+) ssRNA genomes that include the prototypic hypovirus CHV1-EP713, one of the best-studied fungal viruses (Fig. 2C). These viruses were naturally isolated from *C. parasitica* (26, 28), and the standard strain EP155 used in this study was able to support their replication (A. Eusebio-Cope and N. Suzuki, unpublished data). Interestingly, the UTRs of CHV1 and CHV2, both of which have a two-ORF genome structure, had over 9-fold-higher IRES activities than did CHV3 with a single-ORF genome structure. The CHV3 5′ UTR gave still-higher IRES activity than the negative control. The antisense construct of the CHV1 UTR (CHV1-as) showed a similar level of RLU as that of transformants with the empty vector, indicating no IRES activity (Fig. 2D).

**TABLE 1:** Virus sequences tested in this study

Family	Genus	Virus, segment	Abbreviation	Accession no.	Region (nt)	Cistron^a^	IRES activity	Reference
*Hypoviridae*	*Hypovirus*	Cryphonectria hypovirus 1	CHV1	M57938	495 + 72	6	Yes	23
		Cryphonectria hypovirus 2	CHV2	L29010	487 + 72	9	Yes	58
		Cryphonectria hypovirus 3	CHV3	AF188515	369 + 72	4	Yes	59
*Reoviridae*	*Mycoreovirus*	Mycoreovirus 1, S11	MyRV1 S11	AB179643	300 + 72	2	No	18
		Mycoreovirus 3, S10	MyRV3 S10	AB073281	165 + 72	0	No	66
*Partitiviridae*	*Betapartitivirus*	Rosellinia necatrix partitivirus 1, dsRNA2	RnPV1	AB113348	79 + 72	0	No	67
	*Alphapartitivirus*	Rosellinia necatrix partitivirus 2, dsRNA2	RnPV2	AB569998	104 + 72	0	No	68
*Totiviridae*	*Victorivirus*	Helminthosporium victoriae virus 190S	HvV190S	U41345	289 + 72	2	Yes	62
		Rosellinia necatrix victorivirus 1	RnVV1	AB742454	372 + 72	1	Yes	24
*Quadriviridae*	*Quadrivirus*	Rosellinia necatrix quadrivirus 1, dsRNA2	RnQV1	AB620062	106 + 72	0	No	61
*Chrysoviridae*	*Chrysovirus*	Helminthosporium victoriae virus 145S, dsRNA2	HvV145S	AF297177	293 + 72	2	Yes	69
		Cryphonectria nitschkei chrysovirus 1, dsRNA2	CnCV1	DQ865187^b^	215 + 72	2	Yes	60

a Number of minicistrons (over 9 nt, including start and stop codons) in 5′ UTR is shown.

b Partial sequence for CnCV1 dsRNA2; full sequence will be reported elsewhere and is available upon request.

### The CHV1 5′-terminal coding region is important for IRES activity.

**FIG 3:**
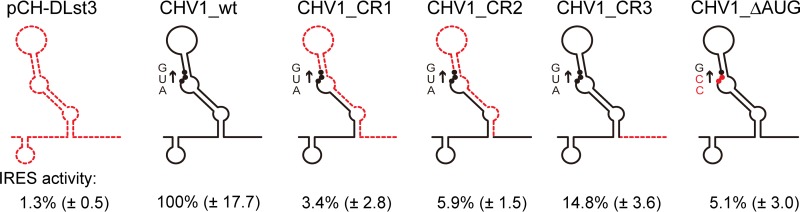
Deletion analysis of CHV1 5′-proximal coding region. The DL assay with 1 s of measurement was conducted using *C. parasitica* transformed with pCH-DLst3 (empty) and its variant carrying the CHV1 5′ UTR (CHV1_wt) without or with deletions/substitution (CHV1_CR1, _CR2, _CR3, or _ΔAUG) in 72 nt from the first ORF. The F/R ratio was calculated as the percentage relative to pCH-DLst3-CHV1 (100%) and numerically presented (values in parentheses are standard deviations). Introduced mutations are schematically represented with a close view of the coding region. Black lines, retained sequence; red dashed lines, deleted regions; black and red dots, AUG start codon and its CCG substitutions, respectively. The expected RNA structure of the CHV1-EP713 5′ terminal region is adopted and depicted based on the work of Mu et al. (53) (see Fig. S3 for the predicted RNA structure of the CHV1 5′ UTR).

Mutational analyses of CHV1 showed the essentiality of the N-terminal 24 codons of the 5′-proximal ORF A (29). Given the fact that the remaining 88% of the coding domain of ORF A is dispensable for virus viability, it is anticipated that these 24 codons are important as an RNA sequence, rather than a part of the p29 protein. However, it remained unknown whether the 24 codons are important for translation or RNA synthesis. In this study, we examined the possibility that the 24-codon region acts as a part of the IRES by using four mutants: three deletion mutants and one substitution mutant as shown in Fig. 3. Deletion of codons 2 to 24 (CHV1_CR1), codons 2 to 12 (CHV1_CR2), and codons 13 to 24 (CHV1_CR3) from the CHV1 IRES foundation construct (CHV1_wt) had detrimental effects on IRES activities (Fig. 3). Furthermore, the substitution of CCG for the first codon, AUG, within the context of CHV1_wt also abolished IRES activity. This indicates that the N-terminal region works as a part of an IRES where the AUG at map position 496 to 498 (codon 1 for the CHV1 ORF A) is utilized in IRES-mediated translation initiation. The results also suggest that in-frame downstream AUG codons in the OFluc coding domain are not used as effective initiators.

10.1128/mBio.02350-17.3 FIG S3  Expected secondary RNA structure of CHV1 5′ terminus. The experimentally predicted RNA secondary structure of the CHV1-EP713 5′ UTR was previously reported by Mu et al. (53); the structure is depicted. Black line, UTR sequences; red line, ORF A coding region; dots, start codon of ORF A. Download FIG S3, TIF file, 0.9 MB. Copyright © 2018 Chiba et al. 2018 Chiba et al. This content is distributed under the terms of the Creative Commons Attribution 4.0 International license.

### Identification of IRESs in diverse fungal dsRNA viruses.

**FIG 4:**
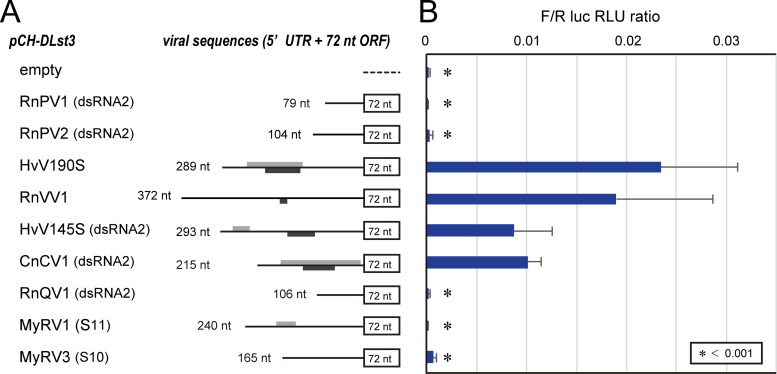
Detection of IRES elements carried by dsRNA mycoviruses. (A) Construction of transformation vectors carrying dsRNA viral cDNA sequences in pCH-DLst3. Lengths of viral 5′ UTRs (nucleotides) and detectable minicistrons over 9 nt are shown as in Fig. 2 (monotone). (B) Dual-luciferase assays. The DL assay was conducted using constructs shown in panel A at 1 s of measurement for both the luciferases. Ratios of OFluc RLU to ORluc RLU (F/R ratios) were calculated and graphically shown. An F/R ratio of less than 0.001 is indicated by an asterisk, representing no IRES activity.

Sequences from dsRNA viruses were then explored. Here, we tested for IRES function in 9 UTRs from 9 different dsRNA viruses that span five dsRNA virus families: *Partitiviridae*, *Totiviridae*, *Chrysoviridae*, *Quadriviridae*, and *Reoviridae* (Table 1). It should be noted that *C. parasitica* supports most tested viruses, except for Cryphonectria nitschkei chrysovirus 1 (CnCV1) (a chrysovirus), Helminthosporium victoriae virus 145S (HvV145S) (a chrysovirus), and Rosellinia necatrix quadrivirus 1 (RnQV1) (a quadrivirus) (24, 30–33). Of the 9 sequences tested (Fig. 4A), IRES activities were detected at differing levels for Rosellinia necatrix victorivirus 1 (RnVV1), CnCV1 dsRNA2, HvV145S dsRNA2, and HvV190S (Fig. 4B). However, no IRES-mediated expression was observed in Rosellinia necatrix partitivirus 1 (RnPV1) dsRNA2, RnPV2 dsRNA2, RnQV1 dsRNA2, mycoreovirus 1 (MyRV1) S11, or MyRV3 S10, and these sequences showed indistinguishable OFluc activities compared to transformants with the empty vector (Fig. 4B).

### Validation of IRES activities using RNA transfection.

**FIG 5:**
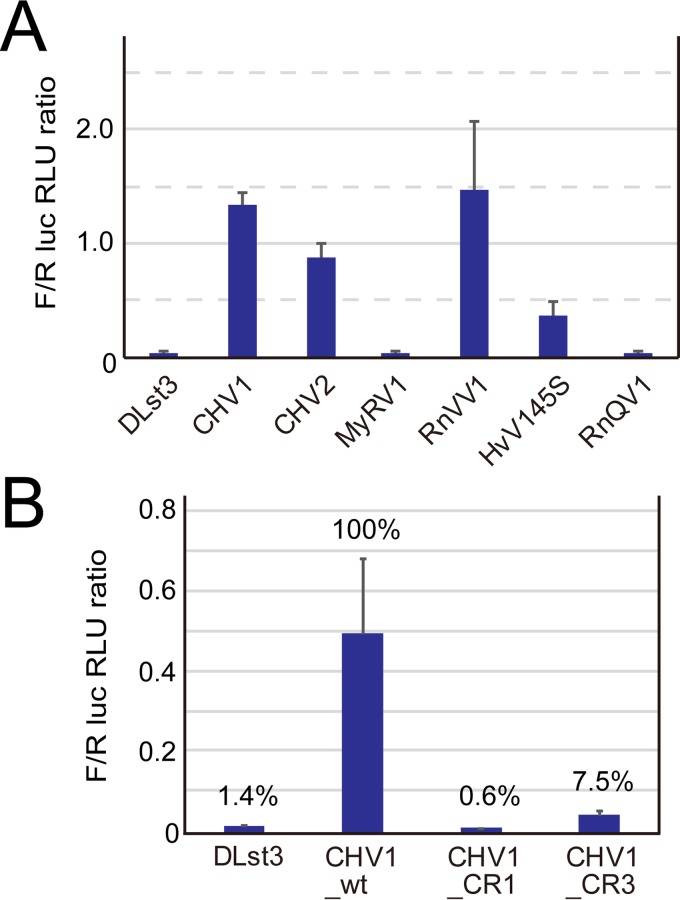
Validation of detected IRESs. (A) Transient IRES assay in fungal protoplasts. *In vitro*-synthesized RNAs carrying DL with the CHV1, CHV2, CHV3, MyRV1, RnVV1, HvV145S, or RnQV1 sequence were introduced into *C. parasitica* cells by electroporation. The DL assay was then conducted at 3 h after electroporation with 12 s of measurement, together with the empty vector (pBS-DLst3) as a negative control. The F/R ratio was calculated, and results from three independent experiments were statistically analyzed as one set of data. (B) IRES activities of CHV1 UTR variants. As performed in panel A, IRES activities were evaluated in the RNA transfection-based transient assay for the constructs CHV1_wt, CHV1_CR1, and CHV1_CR3 together with the empty vector (pBS-DLst3) as a negative control.

A transformation-based assay for IRES activities requires validation because IRES activities detected in such assays may represent those from spliced or degraded transcripts or transcripts from a cryptic promoter with the OFluc ORF situated at the most 5′-proximal end rather than from dicistronic dual-luciferase transcripts (6, 34). To circumvent these problems (35), we took an RNA electroporation approach where *C. parasitica* spheroplasts were transfected with *in vitro*-synthesized RNA from representative constructs. First, the optimal conditions were determined with the transcript carrying the single *OFluc* cistron. The highest OFluc activities were observed when spheroplasts were electroporated at 100 Ω, 2.4 μF, and 0.6 kV or 0.8 kV. These settings were applied to subsequent experiments with representative viral sequences. As shown in Fig. 5A, a pattern of IRES activities indistinguishable from those of transformants was observed. That is, the UTRs of CHV1, CHV2, and RnVV1 had relatively high levels of IRES activity while the HvV145S UTR had low levels of IRES activity. MyRV1 and RnQV1 showed IRES activity similar to the negative control (DLst3) (Fig. 5A).

Another data set for CHV1 and deletion mutant UTRs shown in Fig. 3 was validated by RNA transfection. The same pattern shown by transformants was obtained by an RNA transfection assay; the 5′ CHV1 UTRs lacking 12 or 23 codons (CR3 or CR1, respectively) almost completely lost IRES activities (Fig. 5B), as shown in Fig. 3.

These combined results with RNA transcripts validate the results shown in Fig. 2, 3, and 4 and eliminate the possibility of cryptic promoters or splicing that may affect the conclusions from transformants.

## DISCUSSION

This study has unambiguously demonstrated IRES activities in the 5′ UTRs of diverse fungal RNA viruses, spanning from (+) RNA viruses to dsRNA viruses. This study represents the first report on IRESs originating from filamentous fungi as well as the first report on IRESs from members of certain virus families with RNA genomes such as *Chrysoviridae* and *Hypoviridae*. Also, this study establishes a solid foundation for future mechanistic explorations of required initiation factors, including ITAF and viral *cis* elements.

Our understanding of IRESs has been greatly advanced by studies on IRESs in animal viruses with positive-sense RNA genomes, starting with the discovery of picornavirus IRESs. In contrast, fewer IRESs, whether endogenous or exogenous, have been experimentally demonstrated in fungi, including in the model yeast *S. cerevisiae* (34, 36). The 5′ UTRs of cellular mRNAs of *S. cerevisiae* such as p150 (eIF4G) and TFIID mRNAs were found to be IRES positive (37, 38). The hepatitis C virus (HCV) IRES, a member of type III, and the cricket paralysis virus (CrPV) intergenic IRES, a member of type IV, were shown to function in yeast heterologous systems (39, 40). This heterologous IRES activity may be related to the fact that type III and IV IRESs require only a few host factors. To our knowledge, no endogenous IRESs have been identified in filamentous fungi, for a number of reasons. Generally, *in vitro* and/or *in vivo* tests with bicistronic transforming or transfecting DNA or transfecting RNA are conducted to identify IRESs (6, 34). Single-reporter plasmid DNAs and transcripts with the 5′ end capped or uncapped are also used for measuring IRES activities (41). These constructs occasionally contain stable stem-loop structures upstream of IRES candidates to impair cap-dependent initiation. However, no reporter assay systems suitable for the detection of IRESs or similar translational regulatory elements had been developed in filamentous fungi until recently (27, 42). There are online IRES prediction tools available, like Pfam 12.0 (http://iresite.org/) (43) and IRESPred (http://bioinfo.net.in/IRESPred/) (44). Nevertheless, because of the lack of sequence and structural similarity conserved across different types of IRESs, it is difficult to accurately predict IRESs based solely on their primary sequences (10). In fact, these online IRES prediction tools suggest the presence of IRESs in several mycoviruses such as megabirnaviruses and mycoreoviruses (data not shown), but some of those were not supported by the DL-based experiments in this study, i.e., mycoreoviruses. Thus, validation of the IRES activity of some sequences of interest requires careful biochemical experimentation for each candidate IRES element (6).

Only a few IRES-positive dsRNA viruses are known, such as protozoan-infecting giardiaviruses and leishmaniaviruses (21, 22) within the family *Totiviridae*, which also includes filamentous fungus-infecting victoriviruses and yeast-infecting totiviruses. Viral strains of members in the genus *Totivirus* utilize canonical cap-dependent translation with the cap structures snatched from cellular mRNAs (19). Although some research groups predicted IRESs in their UTRs of members of the genus *Victorivirus* earlier (13), no substantiation was achieved. This study clearly indicates IRES activities in two different victoriviruses and extends the diversity in translation initiation among genera of the family *Totiviridae* (Fig. 4 and 5). The observation that members within a single family, *Totiviridae*, use different translation initiation mechanisms is reminiscent of plant-infecting potyviruses that have different initiation strategies (41).

Hypoviruses with (+) ssRNA genomes were historically discovered as the biological control agents of chestnut blight caused by the chestnut blight fungus, *C. parasitica* (45). There are now four members infecting this fungus (28), and several others have been isolated from other phytopathogenic fungi (46–48). In the current study, all tested representative hypoviruses of *C. parasitica*, i.e., CHV1 to CHV3, were IRES positive. Of note is the finding of an important role of part of the CHV1 coding domain in the IRES. CHV1, one of the best-studied fungal viruses, possesses two contiguous ORFs, A and B. ORF A encodes a multifunctional protein, p29, and a basic protein, p40, while ORF B encodes a large polyprotein carrying a polymerase and a helicase domain (49). Suzuki et al. (29) revealed essential sequence elements located in the first 24 codons of p29 by taking advantage of a reverse genetics system. That is, deletion of the first 12 or 24 codons of ORF A within the context of infectious cDNA causes detrimental effects on virus viability. The authors assumed that the N-terminal coding sequence is required for either translation or RNA synthesis. The results shown in Fig. 3 and 5 strongly suggest that the 5′-terminal region of ORF A (first 24 codons) is part of the CHV1 IRES; thus, its deletion leads to loss of replication competency (29). This situation is similar to the hepatitis C virus (HCV) (type III) IRES, which extends into the 5′ coding region (50–53). Another parallelism is the possible highly structured nature of the CHV1 5′ UTR (53).

Another interesting observation about hypovirus IRESs was that CHV1 and CHV2 had much higher IRES activities than CHV3. There are a few possibilities to account for this difference. It should be noted first that CHV1 and CHV2 are more closely related to each other in their molecular phylogeny and genome organization than to CHV3. CHV1 and CHV2 have double-ORF genomes while the other one has a single-ORF genome. In the two-ORF-type hypoviruses, replication-related proteins encoded on the 3′-proximal portion of the downstream large ORF A are expected to be expressed at relatively lower levels than the ORF A-encoded proteins, because ORF B is translated by the stop/restart mechanism (27). This is generally the case for RNA viruses. Thus, CHV1 and CHV2 might have evolved stronger IRESs after the acquisition of ORF A during the course of evolution. The relatively low IRES activity of CHV3 would avoid unnecessarily excessive expression of replication-related proteins encoded on the 3′-proximal portion of the large ORF. We included the 72-nt coding sequence of CHV3 in the IRES tests as for other viral IRES candidates (Fig. 2 and 4). As another interpretation, full-scale IRES activities of CHV3 may require further internal coding sequences that are beyond the first 24 codons (72 nt) and are not included in the CHV3 construct in this study.

This study establishes a solid foundation for future mechanistic explorations of required initiation factors, including ITAF and viral *cis* elements. Other IRES-positive RNA viruses not discussed above include chrysoviruses. It will be interesting to investigate possible roles of the CAA repeats in the chrysovirus and partitivirus 5′ UTRs, long presumed to be translation enhancers. There are also many other fungal viruses predicted to employ IRES-mediated translation initiation. Such single-stranded, positive-sense fungal viruses include fusariviruses and yadokariviruses, while such dsRNA viruses include megabirnaviruses, yadonushiviruses, botybirnaviruses, and members of the unclassified dsRNA virus groups such as Phlebiopsis gigantea large virus 1 (PgLV1), Circulifer tenellus virus 1 (CiTV1), and Sclerotinia sclerotiorum nonsegmented virus L (SsNsV-L) (54–56). All of these viruses have long 5′ UTRs of over 400 nt and more than one minicistron. Interestingly, all these viruses, along with the abovementioned IRES-positive dsRNA viruses, are members of the expanded *Picornavirus* superfamily (57). Many of the supergroup members are known to have IRES activities (8). Confirmation of the IRES activities of some of these viruses is under way.

## MATERIALS AND METHODS

### Fungal and viral materials.

The standard strain EP155 of the chestnut blight fungus *C. parasitica* was used as an experimental platform for reporter assays. To obtain viral sequences, virus-carrying fungal strains were used: *C. parasitica* strains EP713 (CHV1) (23), NB58 (CHV2) (58), GH2 (CHV3) (59), and 9B21 (MyRV1) (18, 32); *Cryphonectria nitschkei* OB5-11 (CnCV1) (60); *Rosellinia necatrix* strains W8 (RnPV1) (67), W57 (RnPV2) (31), W1029 (RnVV1) (24), and W1075 (RnQV1) (61); and *Helminthosporium victoriae* strain A-9 (HvV190S and HvV145S) (62). Full names and accession numbers of mycoviruses are provided in Table 1. These fungal strains were grown on Difco PDA plates for maintenance and reporter assays.

### Plasmid constructions.

The codon-optimized firefly luciferase gene (*Oluc*), designed for optimal expression in the model filamentous ascomycete *Neurospora crassa*, was kindly provided by J. C. Dunlap (63). The intron sequence was removed, and the BstEII restriction enzyme recognition site was abolished in the original *Oluc* plasmid by the overlap-PCR method to obtain a modified version of codon-optimized firefly luciferase (*OFluc*). The *Renilla* luciferase (*Rluc*) gene was similarly codon optimized (*ORluc*) by *in vitro* nucleotide synthesis and cloned into pUC57-Kan (Genewiz Japan Inc., Saitama, Japan). The original firefly luciferase (*Fluc*) and *Rluc* and codon-optimized *OFluc* and *ORluc* gene fragments were introduced into an expression vector, pCPXHY3, a derivative of pCPXHY1 (64), using HindIII and NotI sites, to obtain pCH-Fluc, -Rluc, -OFluc, and -ORluc, respectively (Fig. 1). To develop the dual-luciferase vector, the *ORluc* and *OFluc* genes were independently PCR amplified and cloned in HindIII and SphI sites of pCPXHY3. A triplet of stop codons (TAATAGTGA) and a multiple cloning site (MCS; 5′-HpaI-BstEII-KpnI-NotI-3′) were designed to be included between 5′-proximal *ORluc* and 3′-proximal *OFluc* (pCH-DLst3) (Fig. 2A and B). All viral cDNAs of the 5′ UTR and adjacent 72-nucleotide (nt) coding domain were amplified by reverse transcription-PCR (RT-PCR) and inserted into the MCS of pCH-DLst3 vector using BstEII and NotI sites, except for the RnVV1 sequence using HpaI instead of BstEII. The antisense sequence of the CHV1 5′ UTR was fused with 72 nt of the coding sequence (sense) by overlapping PCR. Note that these 72 nt and downstream *OFluc* are in frame.

To create the DL transcription vector, pBluescript SK II(+) (pBS) was used. The 3′-terminal sequence of the CHV1 genome containing a poly(A) tail (22 residues) was PCR amplified [200 bp; 5′-HindIII-SphI-PacI-CHV1/poly(A)-SpeI-3′] and inserted in the MCS of pBS vector in the sense orientation to the T7 promoter. The DL cassette from pCH-DLst3 was transferred to this plasmid by using HindIII and SphI recognition sites (pBS-DLst3). Viral sequences were then inserted in pBS-DLst3 using NotI and BstEII or HpaI. These clones were linearized by SpeI and served as the templates for *in vitro* runoff transcription using the RiboMAX kit (Promega).

The sequences of all constructs were confirmed with an ABI3100 sequencer (Applied Biosystems). Oligonucleotide primers used in this study are available upon request. Sequences of *OFluc* and *ORluc* genes are appended as supplemental material (see Fig. S1).

### Luciferase reporter assay using fungal mycelia.

Protoplast isolation, transformation, and regeneration of *C. parasitica* EP155 were performed as described by Faruk et al. (65). The luciferase reporter assays were conducted according to the manufacturer’s instructions (PicaGene Dual [Toyo Ink Group] and dual-luciferase reporter assay system [Promega]). *C. parasitica* transformants were inoculated on cellophane-overlaid PDA plates and cultured for 3 to 4 days on the bench at 22 to 27°C. A small patch of mycelia was obtained using the cap of a 1.5-ml microtube and homogenized in 200 to 500 μl of cell-cracking solution (PicaGene Dual; Toyo Ink Group) in the same tube. Ten microliters of the homogenate was mixed with 100 μl of firefly or *Renilla* luciferase substrate solution in a round-bottom tube (Röhren tube; Sarstedt). Luminescence strength was then measured for 1 to 12 s using a MiniLumat LB 9506 luminometer (Berthold) or a GloMax 20/20 luminometer (Promega). The dual-luciferase reporter assays were conducted by following the manufacturer’s instructions. At least 5 to 10 biological replicates (independent transformants) were analyzed.

### Luciferase reporter assay using fungal protoplasts.

The dual-luciferase reporter assays with the transfection-based transient expression method were conducted as follows. pBS-based bicistronic constructs were linearized by SpeI and served as the templates for *in vitro* runoff transcription using the RiboMAX kit in the presence of the cap analog [5′ 7-methyl guanosine nucleotide, m7G(5′)ppp(5′)G; Promega]. *In vitro*-transcribed DLst3 fragments were introduced into *C. parasitica* protoplasts (1,000,000 cells/100 μl STC [1 M sorbitol, 100 mM CaCl_2_, 100 mM Tris-HCl, pH 8.0] suspension) by electroporation at 0.65 kV, 2.5 μF, and 200 Ω (Gene Pulser electroporation system; Bio-Rad). Liquid RG medium was immediately added to the treated cells, which were placed on ice for 10 min before incubation for 4 h on the bench at 27°C in the dark. Cells were centrifuged (6,000 × *g*, 5 min) and resuspended twice in 1 M sorbitol before final collection and cracking by vortexing in 20 μl of solution (1 part cell-cracking solution and 4 parts 1× phosphate-buffered saline [PBS]). The DL assay was performed as described above with a time count of 12 s. Three independent assays were conducted and statistically analyzed.
