# Supplementary material for: First Evidence for Internal Ribosomal Entry Sites in Diverse Fungal Virus Genomes
Source: mBio. 2018 Mar 20;9(2):e02350-17. doi: 10.1128/mBio.02350-17 (PMC5874917; doi:10.1128/mBio.02350-17)
Supplement: FIG S1 [file mbo002183783sf1.pdf]

**A**

```
         10        20        30        40        50        60
ATGGAGGACGCCAAGAACATCAAGAAGGGCCCCGCCCCCTTCTACCCCCTCGAGGACGGC
M  E  D  A  K  N  I  K  K  G  P  A  P  F  Y  P  L  E  D  G

         70        80        90       100       110       120
ACCGCCGGCGAGCAGCTCCACAAGGCCATGAAGCGCTACGCCCTCGTCCCCGGCACCATC
T  A  G  E  Q  L  H  K  A  M  K  R  Y  A  L  V  P  G  T  I

        130       140       150       160       170       180
GCCTTCACCGACGCCCACATCGAGGTCAACATCACCTACGCCGAGTACTTCGAGATGTCC
A  F  T  D  A  H  I  E  V  N  I  T  Y  A  E  Y  F  E  M  S

        190       200       210       220       230       240
GTCCGCCTCGCCGAGGCCATGAAGCGCTACGGCCTCAACACCAACCACCGCATCGTCGTC
V  R  L  A  E  A  M  K  R  Y  G  L  N  T  N  H  R  I  V  V

        250       260       270       280       290       300
TGCTCCGAGAACTCCCTCCAGTTCTTCATGCCCGTCCTCGGCGCCCTCTTCATCGGCGTC
C  S  E  N  S  L  Q  F  F  M  P  V  L  G  A  L  F  I  G  V

        310       320       330       340       350       360
GCCGTCGCCCCCGCCAACGACATCTACAACGAGCGCGAGCTCCTCAACTCCATGAACATC
A  V  A  P  A  N  D  I  Y  N  E  R  E  L  L  N  S  M  N  I

        370       380       390       400       410       420
TCCCAGCCCACCGTCGTCTTCGTCTCCAAGAAGGGCCTCCAGAAGATCCTCAACGTCCAG
S  Q  P  T  V  V  F  V  S  K  K  G  L  Q  K  I  L  N  V  Q

        430       440       450       460       470       480
AAGAAGCTCCCCATCATCCAGAAGATCATCATCATGGACTCCAAGACCGACTACCAGGGC
K  K  L  P  I  I  Q  K  I  I  I  M  D  S  K  T  D  Y  Q  G

        490       500       510       520       530       540
TTCCAGTCCATGTACACCTTCGTCACCTCCCACCTCCCCCCCGGCTTCAACGAGTACGAC
F  Q  S  M  Y  T  F  V  T  S  H  L  P  P  G  F  N  E  Y  D

        550       560       570       580       590       600
TTCGTCCCCGAGTCCTTCGACCGCGACAAGACCATCGCCCTCATCATGAACTCCTCCGGC
F  V  P  E  S  F  D  R  D  K  T  I  A  L  I  M  N  S  S  G

        610       620       630       640       650       660
TCCACCGGCCTCCCCAAGGGCGTCGCCCTCCCCCACCGCACCGCCTGCGTCCGCTTCTCC
S  T  G  L  P  K  G  V  A  L  P  H  R  T  A  C  V  R  F  S

        670       680       690       700       710       720
CACGCCCGCGACCCCATCTTCGGCAACCAGATCATCCCCGACACCGCCATCCTCTCCGTC
H  A  R  D  P  I  F  G  N  Q  I  I  P  D  T  A  I  L  S  V
```

```
       730       740       750       760       770       780
GTCCCCTTCCACCACGGCTTCGGCATGTTCACCACCCTCGGCTACCTCATCTGCGGCTTC
V  P  F  H  H  G  F  G  M  F  T  T  L  G  Y  L  I  C  G  F

       790       800       810       820       830       840
CGCGTCGTCCTCATGTACCGCTTCGAGGAGGAGCTCTTCCTCCGCTCCCTCCAGGACTAC
R  V  V  L  M  Y  R  F  E  E  E  L  F  L  R  S  L  Q  D  Y

       850       860       870       880       890       900
AAGATCCAGTCCGCCCTCCTCGTCCCCACCCTCTTCTCCTTCTTCGCCAAGTCCACCCTC
K  I  Q  S  A  L  L  V  P  T  L  F  S  F  F  A  K  S  T  L

       910       920       930       940       950       960
ATCGACAAGTACGACCTCTCCAACCTCCACGAGATCGCCTCCGGCGGCGCCCCCCTCTCC
I  D  K  Y  D  L  S  N  L  H  E  I  A  S  G  G  A  P  L  S

       970       980       990      1000      1010      1020
AAGGAGGTCGGCGAGGCCGTCGCCAAGCGCTTCCACCTCCCCGGCATCCGCCAGGGCTAC
K  E  V  G  E  A  V  A  K  R  F  H  L  P  G  I  R  Q  G  Y

      1030      1040      1050      1060      1070      1080
GGCCTCACCGAGACCACCTCCGCCATCCTCATCACCCCCGAGGGCGACGACAAGCCCGGC
G  L  T  E  T  T  S  A  I  L  I  T  P  E  G  D  D  K  P  G

      1090      1100      1110      1120      1130      1140
GCCGTCGGCAAGGTCGTCCCCTTCTTCGAGGCCAAGGTCGTCGACCTCGACACCGGCAAG
A  V  G  K  V  V  P  F  F  E  A  K  V  V  D  L  D  T  G  K

      1150      1160      1170      1180      1190      1200
ACCCTCGGCGTCAACCAGCGCGGCGAGCTCTGCGTCCGCGGCCCCATGATCATGTCCGGC
T  L  G  V  N  Q  R  G  E  L  C  V  R  G  P  M  I  M  S  G

      1210      1220      1230      1240      1250      1260
TACGTCAACAACCCCGAGGCCACCAACGCCCTCATCGACAAGGACGGCTGGCTCCACTCC
Y  V  N  N  P  E  A  T  N  A  L  I  D  K  D  G  W  L  H  S

      1270      1280      1290      1300      1310      1320
GGCGACATCGCCTACTGGGACGAGGACGAGCACTTCTTCATCGTCGACCGCCTCAAGTCC
G  D  I  A  Y  W  D  E  D  E  H  F  F  I  V  D  R  L  K  S

      1330      1340      1350      1360      1370      1380
CTCATCAAGTACAAGGGCTACCAGGTCGCCCCCGCCGAGCTCGAGTCCATCCTCCTCCAG
L  I  K  Y  K  G  Y  Q  V  A  P  A  E  L  E  S  I  L  L  Q

      1390      1400      1410      1420      1430      1440
CACCCCAACATCTTCGACGCCGGCGTCGCCGGCCTCCCCGACGACGACGCCGGCGAGCTC
H  P  N  I  F  D  A  G  V  A  G  L  P  D  D  D  A  G  E  L
```

```
      1450      1460      1470      1480      1490      1500
CCCGCCGCCGTCGTCGTCCTCGAGCACGGCAAGACCATGACCGAGAAGGAGATCGTCGAC
P  A  A  V  V  V  L  E  H  G  K  T  M  T  E  K  E  I  V  D

      1510      1520      1530      1540      1550      1560
TACGTCGCCTCCCAAGTGACCACCGCCAAGAAGCTCCGCGGCGGCGTCGTCTTCGTCGAC
Y  V  A  S  Q  V  T  T  A  K  K  L  R  G  G  V  V  F  V  D

      1570      1580      1590      1600      1610      1620
GAGGTCCCCAAGGGCCTCACCGGCAAGCTCGACGCCCGCAAGATCCGCGAGATCCTCATC
E  V  P  K  G  L  T  G  K  L  D  A  R  K  I  R  E  I  L  I

      1630      1640      1650
AAGGCCAAGAAGGGCGGCAAGTCCAAGCTCTGA
K  A  K  K  G  G  K  S  K  L  *
```

**B**

```
        10        20        30        40        50        60
ATGACCTCCAAGGTCTACGACCCCGAGCAGCGCAAGCGCATGATCACCGGCCCCCAGTGG
M  T  S  K  V  Y  D  P  E  Q  R  K  R  M  I  T  G  P  Q  W

        70        80        90       100       110       120
TGGGCCCGCTGCAAGCAGATGAACGTCCTCGACTCCTTCATCAACTACTACGACTCCGAG
W  A  R  C  K  Q  M  N  V  L  D  S  F  I  N  Y  Y  D  S  E

       130       140       150       160       170       180
AAGCACGCCGAGAACGCCGTCATCTTCCTCCACGGCAACGCCGCCTCCTCCTACCTCTGG
K  H  A  E  N  A  V  I  F  L  H  G  N  A  A  S  S  Y  L  W

       190       200       210       220       230       240
CGCCACGTCGTCCCCCACATCGAGCCCGTCGCCCGCTGCATCATCCCCGACCTCATCGGC
R  H  V  V  P  H  I  E  P  V  A  R  C  I  I  P  D  L  I  G

       250       260       270       280       290       300
ATGGGCAAGTCCGGCAAGTCCGGCAACGGCTCCTACCGCCTCCTCGACCACTACAAGTAC
M  G  K  S  G  K  S  G  N  G  S  Y  R  L  L  D  H  Y  K  Y

       310       320       330       340       350       360
CTCACCGCCTGGTTCGAGCTCCTCAACCTCCCCAAGAAGATCATCTTCGTCGGCCACGAC
L  T  A  W  F  E  L  L  N  L  P  K  K  I  I  F  V  G  H  D

       370       380       390       400       410       420
TGGGGCGCCTGCCTCGCCTTCCACTACTCCTACGAGCACCAGGACAAGATCAAGGCCATC
W  G  A  C  L  A  F  H  Y  S  Y  E  H  Q  D  K  I  K  A  I
```

```
        430       440       450       460       470       480
GTCCACGCCGAGTCCGTCGTCGACGTCATCGAGTCCTGGGACGAGTGGCCCGACATCGAG
V  H  A  E  S  V  V  D  V  I  E  S  W  D  E  W  P  D  I  E

        490       500       510       520       530       540
GAGGACATCGCCCTCATCAAGTCCGAGGAGGGCGAGAAGATGGTCCTCGAGAACAACTTC
E  D  I  A  L  I  K  S  E  E  G  E  K  M  V  L  E  N  N  F

        550       560       570       580       590       600
TTCGTCGAGACCATGCTCCCCTCCAAGATCATGCGCAAGCTCGAGCCCGAGGAGTTCGCC
F  V  E  T  M  L  P  S  K  I  M  R  K  L  E  P  E  E  F  A

        610       620       630       640       650       660
GCCTACCTCGAGCCCTTCAAGGAGAAGGGCGAGGTCCGCCGCCCCACCCTCTCCTGGCCC
A  Y  L  E  P  F  K  E  K  G  E  V  R  R  P  T  L  S  W  P

        670       680       690       700       710       720
CGCGAGATCCCCCTCGTCAAGGGCGGCAAGCCCGACGTCGTCCAGATCGTCCGCAACTAC
R  E  I  P  L  V  K  G  G  K  P  D  V  V  Q  I  V  R  N  Y

        730       740       750       760       770       780
AACGCCTACCTCCGCGCCTCCGACGACCTCCCCAAGATGTTCATCGAGTCCGACCCCGGC
N  A  Y  L  R  A  S  D  D  L  P  K  M  F  I  E  S  D  P  G

        790       800       810       820       830       840
TTCTTCTCCAACGCCATCGTCGAGGGCGCCAAGAAGTTCCCCAACACCGAGTTCGTCAAG
F  F  S  N  A  I  V  E  G  A  K  K  F  P  N  T  E  F  V  K

        850       860       870       880       890       900
GTCAAGGGCCTCCACTTCTCCCAGGAGGACGCCCCCGACGAGATGGGCAAGTACATCAAG
V  K  G  L  H  F  S  Q  E  D  A  P  D  E  M  G  K  Y  I  K

        910       920       930       940
TCCTTCGTCGAGCGCGTCCTCAAGAACGAGCAGTAATAGTGA
S  F  V  E  R  V  L  K  N  E  Q  *  *  *
```
