# Supplementary figures and images for: First Evidence for Internal Ribosomal Entry Sites in Diverse Fungal Virus Genomes
Source: mBio. 2018 Mar 20;9(2):e02350-17. doi: 10.1128/mBio.02350-17 (PMC5874917; doi:10.1128/mBio.02350-17)

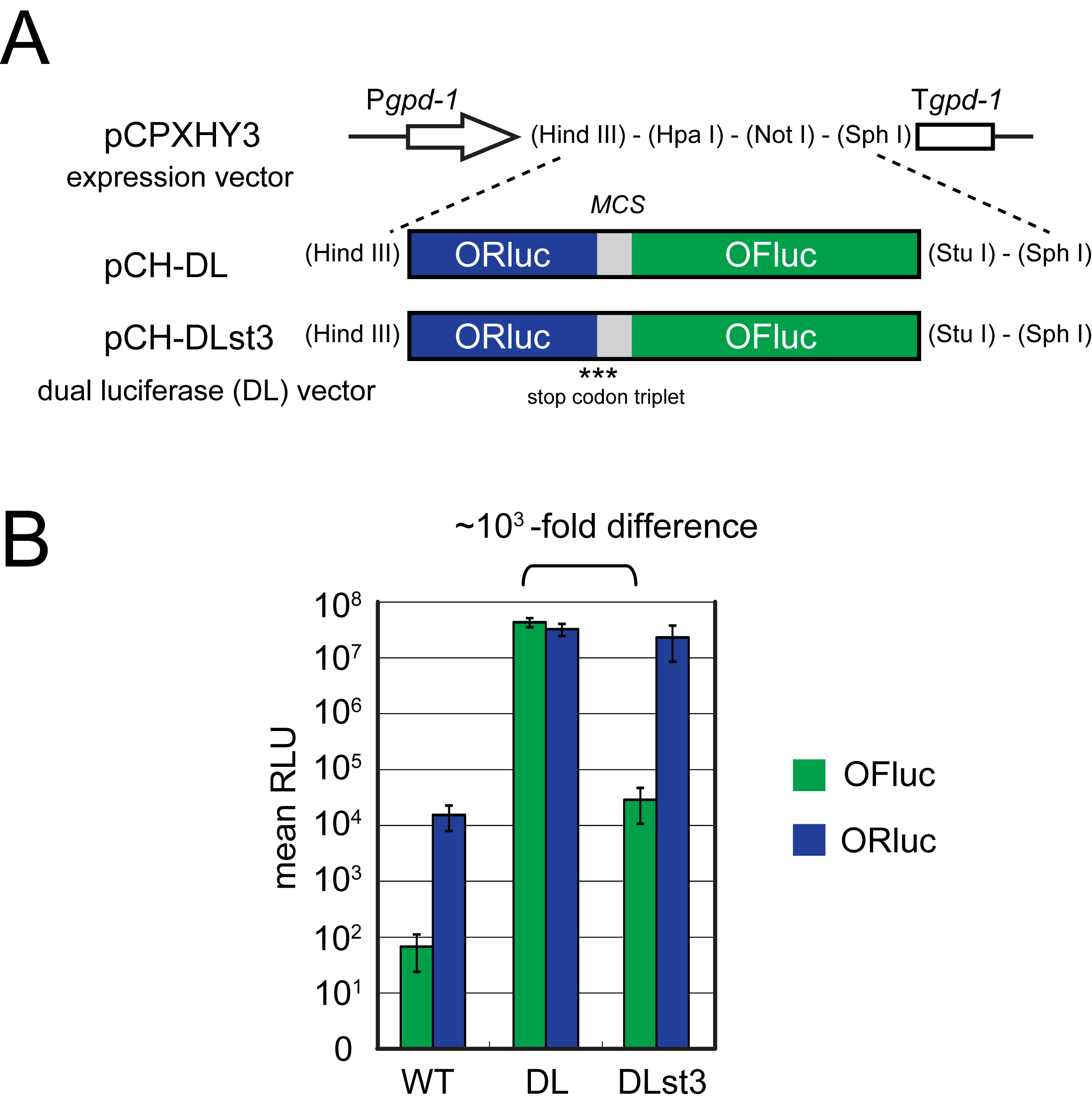

Supplement: FIG S2 [file mbo002183783sf2.tif]

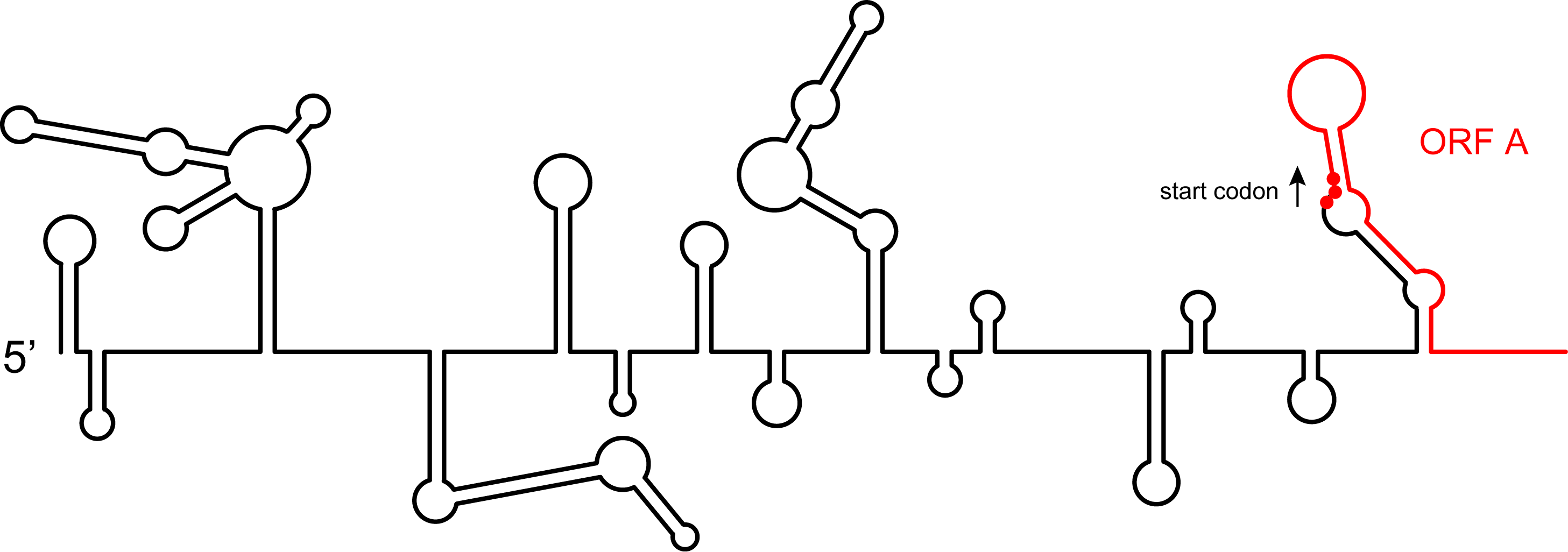

Supplement: FIG S3 [file mbo002183783sf3.tif]
